# The constructive nature of memories in insects: bumblebees as a case study

**DOI:** 10.1098/rstb.2023.0405

**Published:** 2024-09-16

**Authors:** Gema Martin-Ordas

**Affiliations:** ^1^ Division of Psychology, University of Stirling, Stirling FK9 4LA, UK

**Keywords:** memory conjunction errors, constructive memory, episodic memory, wild bumblebees, animals

## Abstract

The view that human memory is constructive implies that recollections are not necessarily an accurate reproduction of past events. An approach to study this constructive nature of memory is by examining memory errors. In this regard, conjunction errors—i.e. incorrect recollection of new stimuli integrated by components from two previously studied stimuli—have attracted important attention in human memory research. Do animals other than humans make conjunction errors? To investigate this issue, a choice task in which training was not involved was used. Bees experienced two to-be-remembered stimuli. At the test, they were presented with four stimuli: one of the original items (i.e. old), an item made by combining two features of the original items (i.e. conjunction), an item containing a previously presented feature and a new one (i.e. feature), and an item integrated solely by new features (i.e. new). Bumblebees remembered the old items. Importantly, when making memory errors, bumblebees selected conjunction and feature lures more often than new items. These results indicate that bumblebees, like humans, spontaneously make memory conjunction errors and suggest that invertebrates’ memories might also be constructive in nature. I suggest that focusing on memory errors is a solid avenue to investigate episodic (like) memory in animals.

This article is part of the theme issue ‘Elements of episodic memory: lessons from 40 years of research’.

## 1. Introduction

In the opening paragraph of *Elements of Episodic Memory*, Tulving [1] wrote, ‘Remembering past events is a universally familiar experience. It is also a uniquely human one. As far as we know, members of no other species possess quite the same ability to experience again now, in a different situation and perhaps in a different form, happenings from the past, and know that the experience refers to an event that occurred in another time and in another place’ (p. 1). An important aspect of Tulving’s definition of episodic memory was the phenomenological experience attached to the content of the memory [1]. Specifically, Tulving considered autonoetic awareness—i.e. a type of consciousness that allows the owner of a memory to recollect a past event as an event that happened to the owner in the past—to be a critical feature that allowed distinguishing episodic from semantic memory. Both episodic (e.g. I remember where I went last weekend) and semantic (e.g. I know when and where I was born) memories could involve recollecting spatio-temporal information. However, the latter does not require any re-experience (i.e. autonoetic awareness) of the past event [1–3]. Tulving’s emphasis on the phenomenological aspect of episodic memory means that behavioural outcomes alone are not sufficient to demonstrate whether non-human animals (and preverbal children) can remember personal past events. This is because language is usually needed to establish autonoetic awareness.

Although 30 years later it is still under debate whether episodic memory is uniquely human, comparative researchers have made important efforts in developing creative experimental paradigms to investigate this ability in non-human animals (henceforth, animals). Three main conceptualizations have been used to test episodic memory in animals (e.g. [4]): (i) *Episodic memory involves remembering the spatial–temporal components of a past event*—so-called memory for what-where-when or episodic-like memory (e.g. [5]). Note that, as defined by Clayton and colleagues (e.g. [6,7]), the binding of the components integrating an event is required for this type of recollection (see also [8,9]); (ii) *Episodic recollection can be distinguished from familiarity, which is associated with semantic memory* (e.g. [10]). Whereas the latter does not involve the retrieval of the event in which an item was experienced, recollection does [11,12]; (iii) *Episodic memory contributes to future thinking* (e.g. [13]). It was not until later in his writings that Tulving (e.g. [2,3,14]) described episodic memory to be a crucial part of a system adapted for thinking about the past, present and future—so-called mental time travel (MTT) [15]. Specifically, Tulving [14] wrote that ‘this mental time travel allows one, as an “owner” of episodic memory (“self”), through the medium of autonoetic awareness, to remember one’s own previous “thought-about” experiences, as well as to “think about” one’s own possible future experiences’ (p. 9). Thus, it is autonoetic awareness that makes possible mentally travelling in time.

But how does episodic memory contribute to future thinking? In his very first definition of episodic memory, Tulving [16] suggested that this type of memory was, to some degree, an accurate record of the ‘owner’s’ experiences. Later elaborations of the concept, however, indicated a nuanced change in Tulving’s understanding of episodic memory and that ‘although a good deal of remembering is more or less veridical, a good deal of it is not’ [1, p. 187]. This later perspective is well aligned with more recent conceptions of episodic memory that stress the importance of its constructive nature [17–20]. Considering episodic memory as a construction capacity allows understanding future thinking as an expression of the former. This is because construction processes are at play in joining pieces of episodic information when thinking about and imagining future events [20–22].

A consequence of relying on constructive processes when remembering past events is that recollection is susceptible to errors [23]. Episodic memories are stored as being integrated by different fragments that, at retrieval, have to be relocated and bound together (e.g. [24]). However, the binding may fail, therefore generating memory errors. For example, the details of two different memories from a list of to-be-remembered words (e.g. ‘blacktop’, ‘headboard’) can sometimes be incorrectly bound and remembered as a true memory (e.g. we recollect ‘blackboard’)—so-called memory conjunction errors (e.g. [25–27]). These constructive memory processes have been rarely investigated in animals (e.g. [28–31]), and they offer a new approach to investigating episodic memory—and MTT, more generally—by providing critical evidence for the episodicity of memory. Thus, an approach to understanding episodic memory and its constructive nature is to examine the occurrence of memory errors.

Here, I will examine whether wild-caught bumblebees are susceptible to memory conjunction errors. In humans, these effects have been described to occur for words and pseudowords [25,32,33], two-word phrases (e.g. *last week*; [33]), faces (e.g. [26,27]) and personal past events (e.g. [34]). Importantly, memory conjunction errors have also been associated with phenomenological experiences—which suggests a certain similarity with true episodic memories (e.g. [26,35]). Bumblebees have extraordinary spatial memory skills (e.g. [36,37]) and have been shown to erroneously merge features of previously experienced flowers when trained to remember these features [38]. Thus, they are excellent candidates to investigate the constructive nature of their memories. In two experiments, bees were presented first with to-be-remembered stimuli and next with a choice task involving four stimuli (old, conjunction, feature and new). Conjunction lures consisted entirely of previously presented old stimuli recombined to create a novel item. Feature lures consisted of part of a previously presented old stimulus combined with a new feature. New stimuli were integrated with features not presented before to the bees. With this experimental approach, it is plausible to examine whether bees spontaneously make memory conjunction errors. It was hypothesized that bees will show higher levels of false alarm responses to stimuli integrated with previously seen features, if construction processes underlie their memories.

## 2. Experiment 1: spontaneous memory conjunction

**Figure 1.**
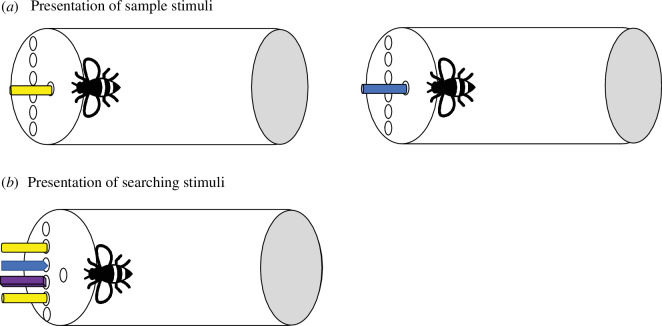
Bird’s eye view of the experimental set-up for experiment 1. Bees experience the two sample stimuli in the sample hole (*a*). The sample stimuli were dipped in sucrose. After 1 min, bees are presented with the searching stimuli in the searching array. From top to the bottom, the schema depicts the *old* stimulus, *feature* lure, *new* stimulus and *conjunction* lure (*b*). Note that the darker borders in the stimuli have only been used to represent the different shapes of the items in the drawing.

Bumblebees experienced two sets of objects (sample and searching arrays). First, bees were sequentially presented with two to-be-remembered objects—both were dipped in sucrose. After 1 min retention interval (RI), bees were presented with four stimuli (*old*, *conjunction*, *feature* and *new*). Of interest was what stimulus was selected first (see figure 1).

### (a) Subjects

The data were collected in June and July 2022 in Stirlingshire (UK). A total of 25 bees were captured, and they all completed at least three out of the four trials. Note that 22 bees completed all the trials, and three bees completed three trials. The final sample was integrated by 25 bees of the following species: *Bombus pascuorum* (*n* = 8), *B. terrestris complex* (*n* = 14) and *B. hypnorum* (*n* = 3). Sex was visually identified (females = 25), and no queens were tested.

### (b) Apparatus

A transparent plastic tube (11 × 4.5 cm) with seven holes through which the stimuli could be inserted was used (see figure 1). A bottom set of holes—searching array—consisted of six holes with 4 mm between them. Above the set of holes, a single hole was drilled—sample hole—and the distance between this hole and the middle bottom ones was 5 mm. The following items were used as stimuli: blue paper strips (3 × 0.2 cm), orange paper strips (3 × 0.2 cm), yellow paper strips (3 × 0.2 cm), blue plastic strips (3 × 0.1 cm), orange plastic strips (3 × 0.1 cm), yellow plastic strips (3 × 0.1 cm), purple plastic strips (3 × 0.1 cm), blue paper lollipops (3 × 0.4 cm), yellow paper lollipops (3 × 0.4 cm), purple paper lollipops (3 × 0.4 cm), orange wooden sticks (3 × 0.2 cm), blue wooden sticks (3 × 0.2 cm) and purple wooden sticks (3 × 0.2 cm). These stimuli differed in colour and shape (paper strips: flat with a rectangular shape ending; plastic strips: flat with triangle shape ending; paper lollipops: rounded; wooden sticks: cubical). Two stimuli, one after the other, were introduced through the sample hole. Four stimuli, fixed in playdoh, were introduced simultaneously through the searching array.

### (c) Procedure

Experiments were always conducted between 07.30 and 10.00. Subjects were left in the tube on average for 1 h prior to testing to allow them to habituate to the tubes and become motivated to forage [39]. Bees were caught directly from flowers by using the testing tubes in which the experiments were conducted. This minimized the manipulation of the bees. For each trial, bees experienced two sample stimuli and four testing stimuli. Subjects were first presented with the sample stimuli in the sample hole. These two stimuli were presented to the bees sequentially, and both were dipped in 50% (w/w) sucrose. Once the bee made contact with the first sample stimulus (e.g. yellow paper strip)—either by using its antennae or proboscis—it was given (on average) 5–6 s to drink the solution. Then, the stimulus was removed. The same procedure was repeated for the second sample stimulus (e.g. blue lollipop). Next, and after a retention interval (RI) of 1 min, the experimenter (E) introduced the searching strips (e.g. old: yellow paper strip; conjunction: yellow lollipop; feature: blue plastic strip; new: purple wood) through the searching array when the bees were not near the stimuli (i.e. 1.5–2 cm away from the stimuli). These four stimuli were dipped in water. The location of old, conjunction, feature and new stimuli was randomized across trials and subjects. The first stimulus that bees touched with the antennae or proboscis was considered a choice.

Each bee received a total of four trials. The same four pairs of stimuli were used as sample (one pair per trial) across subjects: (i) yellow lollipop and orange paper strip, (ii) yellow paper strip and blue lollipop, (iii) blue wooden stick and purple plastic strip, and (iv) orange plastic strip and purple wooden stick. The order in which these sample stimuli were presented was randomized across subjects. Within each pair, which sample stimulus was presented first was also randomized across subjects. For each subject, in 50% of the trials, the old item was the first sample stimulus, and in the other 50%, the old item was the second sample stimulus. The conjunction stimulus was integrated by two previously experienced features—either colour or shape from the first sample stimuli and either colour or shape of the second sample stimulus. The feature stimulus consisted of the colour of sample stimulus not used as an old stimulus and a shape not experienced in the sample stimuli. Note that in two trials the shape of the old stimulus and a colour not experienced in the sample stimuli were integrated into the feature lure. The new stimuli were integrated by a colour and shape not experienced in the sample stimuli. Thus, the conjunction stimuli shared two features with the sample stimuli, the feature stimuli shared one feature with the sample stimuli and the new stimuli shared no features with the sample stimuli. New materials (i.e. sample and searching stimuli) were used in each trial. The inter-trial-intervals were approximately 2 min for each bee. During this time, subjects were allowed to freely move in the tube. Importantly, bees did not receive any training prior to these trials and their choices were not rewarded.

### (d) Analyses

Data were analysed using R version 03.0+386 using a binomial general linear mixed model (GLMM) [40]. The dependent variable was bees’ choices. Specifically, for each trial, bees’ first selected stimulus was coded as 1 and the non-selected ones as 0. To compare the different groups, type of stimulus (i.e. old, conjunction, feature and new) as a categorical variable was used as independent variable. The following were included as random factors: individual bees, trial number, order presentation of the to-be-remembered stimuli, and the to-be-remembered stimuli. Six models were run to analyse the differences between each type of stimulus. Another model was run by adding species as a random factor and the results remained unchanged (see the electronic supplementary material). Wilcoxon tests were used to analyse if performance was significantly above chance. Values of *p* < 0.050 were considered to provide evidence for significant differences.

### (e) Results and discussion

**Figure 2.**
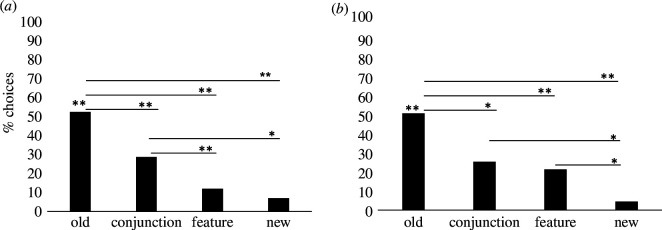
(*a*) represents the mean percentage of bees’ first choices of each stimulus in experiment 1. Here subjects were presented with two to-be-remembered sample stimuli, and, at the test, they were asked to choose between a previously presented stimulus (*old*) and three novel ones that varied in the number of features shared with the sample stimuli (*conjunction*: two features shared with the sample stimuli; *feature*: one feature shared with the sample stimuli; *new*: no features shared with the sample stimuli). (*b*) represents the mean percentage of bees’ first choices of each stimulus in experiment 2. In this experiment, bees were presented with four sample stimuli (the first two were introduced through the sample hole, and the last two stimuli were placed far enough from the tube so bees could see them but not touch them). As in experiment 1, at the test, bees were asked to choose between a previously presented stimulus (*old*) and three novel ones that varied in the number of features shared with the sample stimuli (*conjunction*: two features shared with the sample stimuli; *feature*: one feature shared with the sample stimuli; *new*: no features shared with the sample stimuli). The asterisks indicate *p < 0.05, **p < 0.001.

Bees correctly selected the *old* stimulus significantly above chance (*W* = 185, *p *< 0.001). When the bees’ first choices across the four trials were analysed, it was found that their choices differed across the type of stimuli. In particular, bees selected the *old* stimuli more often than *conjunction* (estimate s.d. = −0.964, *z* = −3.19, *p* = 0.001, 95% CI = 0.210 to 0.690), *feature* (estimate s.d. = −2.020, *z* = −5.47, *p *< 0.001, 95% CI = 0.064 to 0.274) and *new* stimuli (estimate s.d. = −2.616, *z* = −5.92, *p *< 0.001, 95% CI = 0.030 to 0.174; see figure 2*a*). Bees also chose the *conjunction* stimulus first more often than *feature* (estimate s.d. = −1.056, *z* = −2.77, *p* = 0.006, 95% CI = 0.164 to 0.734) and *new* stimuli (estimate s.d. = −1.652, *z* = −3.66, *p *< 0.001, 95% CI = 0.079 to 0.465). No differences between *feature* and *new* stimuli were found (estimate s.d. = −0.596, *z* = −1.19, *p* = 0.232, 95% CI = 0.207 to 1.465; see figure 2*a*).

These results indicate that, in a choice task, bumblebees spontaneously show higher levels of false recognition for conjunction lures compared with features and new stimuli. Importantly, feature lures were not selected more often than new stimuli. With some exceptions (e.g. [26]), familiarity with the previously studied items is considered to account for memory conjunction errors (e.g. [41–43]). This is because, for example, conjunction lures (integrated by two old features) possess a higher familiarity strength than feature (integrated by one old feature) and new lures. However, if familiarity were to account for the present findings, then feature lures should have been selected more often than new stimuli. The failure to find a difference suggests that a familiarity-based explanation cannot account for these results. Note, though, that for each trial the colour of the to-be-remembered items appeared twice either in the old item and conjunction lure or in the conjunction and feature lures. The colour could have been a salient feature of the stimuli, which might have facilitated bees’ performance by only remembering the colour of the to-be-remembered items. The fact that bees were significantly above chance at selecting the old item speaks against this possibility. However, experiment 2 controlled for this issue. In addition, experiment 2 also examined whether bumblebees would choose more frequently, at least, conjunction lures, when sample stimuli were not directly associated with a reward. This manipulation allowed for a more direct comparison with the human studies, in which participants are not rewarded for studying the to-be-remembered stimuli.

## 3. Experiment 2: unrewarded sample stimuli

The same set-up as in experiment 1 was used, although with three critical differences: (i) bees were now presented with four sample stimuli. The first two were rewarded, and the last two were not. One of the non-rewarded stimuli was used to test bees’ memories; (ii) each of the four searching stimuli was of a different colour. This allowed us to control for the effect that the salience of the colours might have on bees’ memories; (iii) the same sample and searching stimuli were used in two trials. This manipulation allowed us to further examine the role that familiarity plays in bees’ memory errors. Research in humans has shown that study repetition increases familiarity and, consequently, feature and conjunction effects [44–46]. Thus, if, like in humans, repetition increases familiarity, bees were expected to make more false recognitions for *conjunction* and *feature* lures in the second trial compared with the first one.

### (a) Subjects

The data were collected in July and August 2022 in Stirlingshire (UK). A total of 34 bees were captured—with 33 bees completing the two trials and one bee completing one trial. The final sample was integrated by the following species: *B. terrestris complex* (*n* = 19), *B. pascuorum* (*n* = 10), *B. bohemicus* (*n* = 2), *B. hortorum* (*n* = 2) and *B. hypnorum* (*n* = 1). Sex was visually identified (females = 34), and no queens were tested.

### (b) Apparatus

The same tubes as in experiment 1 were used. The following were used as stimuli: white plastic strips (3 × 0.1 cm), red plastic strips (3 × 0.1 cm), blue paper lollipops (3 × 0.4 cm), yellow paper lollipops (3 × 0.4 cm), orange paper strips (3 × 0.2 cm), blue paper strips (3 × 0.2 cm) and purple pipe cleaner (3 × 0.5 cm). These stimuli differed in colour and shape (paper strips: flat with a rectangular shape ending; plastic strips: flat with a triangle shape ending; paper lollipops and pipe cleaner: rounded).

### (c) Procedure

**Figure 3.**
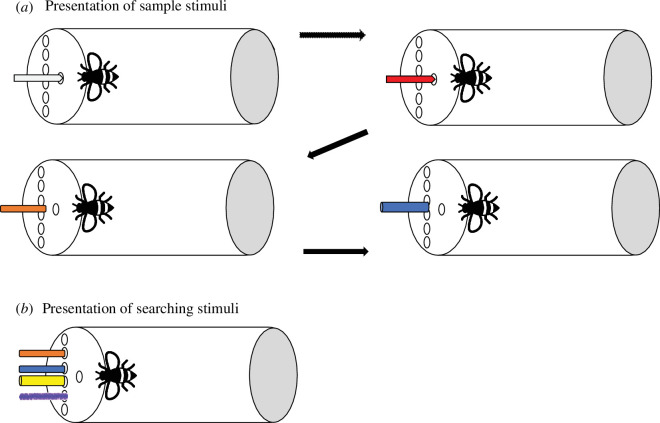
Bird’s eye view of the experimental set-up for experiment 2. Bees experience the four sample stimuli in the sample hole (*a*). The first two stimuli were dipped in sucrose and were reachable to the bees. The last two could not be reached by the bees. After 1 min, bees are presented with the searching stimuli in the searching array. From top to the bottom, the schema depicts the *old* stimulus, *conjunction* lure, *feature* lure and *new* stimulus (*b*). Note that the darker borders in the stimuli have only been used to represent the different shapes of the items in the drawing.

Bees received two trials and, in both, the same sample and searching stimuli were presented (see figure 3). The bees experienced four sample stimuli: the first two (white plastic strip and red plastic strip) were dipped in 50% (w/w) sucrose and the other two stimuli (orange paper strip and blue lollipop) were not. The first two sample stimuli were introduced one after the other through the sample hole. The other two were shown one after the other and were left for an average of 6 s next to the sample hold, but far enough so bees could not reach them and, thus, could not touch them. This two-step sample presentation was done for bees to learn that the stimuli presented through the sample hole had sucrose. Importantly, the two to-be-remembered stimuli—orange paper strip and blue lollipop—were never directly rewarded. The orange paper strip was always used as old. For each subject, the orange paper strip was presented third in one of the trials and fourth in the other. This order was randomized across subjects. The conjunction lure (i.e. blue paper strip) was integrated by the colour of the other non-rewarded sample stimulus (i.e. blue paper lollipop) and the shape of the old stimulus (i.e. flat strip). The feature lure (i.e. yellow paper lollipop) was integrated by the shape of the non-rewarded stimulus (i.e. lollipop paper stick) and a new colour (i.e. yellow) not experienced during the presentation of the sample stimuli. The new stimulus consisted of a shape/material and colour not presented before to the bees (i.e. purple pipe cleaner). Thus, the conjunction stimulus shared two features with the non-rewarding sample stimuli, the feature stimulus shared one feature with the non-rewarding sample stimuli and the new stimulus shared no features with the non-rewarding sample stimuli. New materials were used in each trial. As in experiment 1, the inter-trial-intervals were approximately 2 min for each bee, bees did not receive any training prior to these trials and their choices were not rewarded.

### (d) Analyses

As in experiment 1, I used a binomial GLMM to analyse bees’ choices, with type of stimulus (i.e. *old*, *conjunction*, *feature* and *new*) being the independent variable and individual bees, trial number and order presentation of the to-be-remembered stimuli being the random factors. Six models were run to analyse the differences between each type of stimulus. An additional model was run by adding species as a random factor, and the results remained unchanged (see the electronic supplementary material). Finally, a model was run with the dependent variable being whether bees’ choices were correct (i.e. selected old stimulus, coded 1) or incorrect (i.e. selected any other stimuli, coded 0). The fixed factor was the trial number as a categorical variable and the random factor was individual bees. Given that the number of errors was low, this second model was done to examine the effect of repetition on bees’ choices. Binomial tests were used to evaluate if bees’ performance was significantly above chance. Values of *p* < 0.050 were considered to provide evidence for significant differences.

### (e) Results and discussion

When bees’ first choices across the two trials were analysed (figure 2*b*), it was found that bees selected the *old* stimuli more often than *conjunction* (estimate s.d. = −1.05, *z* = −2.82, *p* = 0.005, 95% CI = 0.168 to 0.727), *feature* (estimate s.d. = −1.30, *z* = −3.36, *p *< 0.001, 95% CI = 0.127 to 0.581) and *new* stimuli (estimate s.d. = −3.03, *z* = −4.74, *p *< 0.001, 95% CI = 0.013 to 0.169). Bees did not select *conjunction* lures more often than *feature* stimuli (estimate s.d. = −0.252, *z* = −0.614, *p* = 0.539, 95% CI = 0.347 to 1.739), but they did so compared with *new* stimuli (estimate s.d. = −1.981, *z* = −3.030, *p* = 0.002, 95% CI = 0.038 to 0.497). Finally, bees selected first *feature* lures more frequently than they selected *new* ones (estimate s.d. = −1.729, *z* = −2.609, *p* = 0.009, 95% CI = 0.048 to 0.650). Bees correctly selected the *old* stimulus significantly above chance in both trials (binomial tests, trial 1: *p *< 0.001; trial 2: *p* = 0.027), and no difference in bees’ performance was found between Trials 1 and 2 (estimate s.d. = −0.418, *z* = −0.852, *p* = 0.394, 95% CI = 0.251 to 1.72).

Like in experiment 1, bees remembered the old stimuli. They also showed higher levels of false recognition for conjunction and feature lures compared with new stimuli. In contrast to experiment 1, however, their choices between conjunction and feature lures did not differ. Since bees’ choices did not differ between stimuli integrated by different numbers of old features, it is challenging to argue that familiarity can account for these results. Moreover, repetition did not diminish bees’ memories of the old stimulus. This is in contrast to what has been shown in humans. Note, though, that some studies demonstrate that humans can be better at rejecting lures with repeated presentations of the same stimuli (e.g. [47–49]). However, bees’ performance did not improve in the second trial either. To examine the effect of repetition in humans, particular items are presented several times during the encoding phase (e.g. [41]). In the present study, however, bees were presented twice with the to-be-remembered items, but their memory was assessed before (trial 1) and after (trial 2) the repetition. Thus, this methodological departure from standard procedures could explain why repetition did not affect bees’ performance.

## 4. General discussion

Experiments 1 and 2 showed that, in a choice task, wild-caught bumblebees remembered the items presented as sample stimuli. When making memory errors, bumblebees consistently selected *conjunction* lures (experiment 1) and *feature* lures (experiments 1 and 2) more often than *new* items. Importantly, bees did so without receiving any training with the to-be-remembered items (figure 3).

These results replicate and extend previous findings on memory errors in bumblebees [38]. Specifically, bees discriminated between old stimuli and conjunction lures—indicating that they differentiated old stimuli from novel stimuli integrated by two old features (experiments 1 and 2). Second, bees also discriminated between conjunction lures and stimuli that consisted of one new feature (i.e. feature lures; experiment 1) and two new features (i.e. new; experiments 1 and 2). Importantly, the conjunction and feature errors occurred after short retention intervals, regardless of which one of the two sample stimuli was tested and which features of the sample stimuli were included in the lures.

Experiment 2 also showed that bees remembered an item that was never directly rewarded. Remarkably, bees’ memories for the non-rewarded items still led them to choose the old stimuli, as in experiment 1 (i.e. when both sample stimuli were associated with a reward). That is, not only the salience of the rewarded items did not overtake bees’ ability to encode and remember the non-rewarded items, but also, when making errors, bees incorrectly identified items integrated by old features as old. Thus, bees make memory conjunction and feature errors that cannot be explained by the salience of the association with a reward [50].

As already mentioned, in humans, the commonly accepted explanation for memory conjunction errors assumes that familiar items are more probable to be identified as *old*. In this regard, since *conjunction* lures are integrated by two features from two previously studied items, they feel familiar and, therefore, prompt false alarms [44,45,51]. *Feature* lures share only one feature with the studied item and, thus, yield fewer errors. *New* items do not share any features with the studied items; therefore, they trigger the lowest error rate. This familiarity account cannot fully explain bees’ performance in the present studies. The critical piece of evidence is that bees did not select *feature* lures more often than *new* ones (experiment 1) nor *conjunction* more frequently than *feature* lures (experiment 2). Moreover, repetition did not influence bees’ choices in experiment 2. Besides the procedural differences with the human literature, this finding could also be explained by the fact that repetition has been reported to have two effects [48]. On the one hand, repetition increases familiarity, prompting false recognition, and, on the other, repetition increases memory for the to-be-remembered items, reducing false recognition. These opposing effects could have led to a lack of effect from repetition on *conjunction* errors [52–54].

Thus, it is arguable that in the present tasks, bees failed to encode the global configuration of the sample stimuli (e.g. orange cubical stick, yellow flat strip; [26]). Accordingly, bees would have encoded the features of the stimuli (e.g. orange cubical and yellow flat) independently and, then, bound to form a configuration of the stimuli (e.g. orange flat strip). Importantly, at the test, bees would have retrieved the features separately (e.g. orange flat strip) and joined together in the process of retrieving then. This process would have created the impression that the conjunction lure was presented as a sample stimulus. Under this approach, recollection contributes to producing conjunction errors. Jones & Jacoby [44] argue, though, that it is precisely recollection that prevents conjunction and feature errors from occurring. It is only old stimuli that are correctly identified via recollection. While it is not possible to irrefutably establish recollection as the process underlying bees’ performance in the present studies, it is also true that familiarity alone is not well supported by the current results.

Previous research has shown that bees are sensitive to shape and colour of the flowers [55], and a recent study using a similar task to the one used here has shown that bees bind shape and colour into their representation of objects [56]. Thus, it is plausible that bees perceived and remembered the features (e.g. colour and shape) that integrate the sample stimuli. The fact that bees succeeded at remembering the old items supports this claim—particularly in experiment 1 when feature and conjunction lures shared colour with the sample stimuli.

What is the significance of studying memory errors in bumblebees? In humans, the recombination processes that are critical for memory recollection are argued to make memory prone to errors that arise from mistakenly combining elements of stored episodes [1,14,23,57]. In this context, memory conjunction errors [26] are usual forms of memory distortions [58,59]. If conjunction mistakes made by bees in the present studies indeed arise from erroneously merging elements of the to-be-remembered items, then one would be tempted to conclude that bees’ memories are also constructive. It is completely plausible to expect that these types of errors are present in bees because their natural lifestyle involves encoding and retrieving features from several stimuli (e.g. flowers). More evidence is needed before this conclusion can be reached, though [60]. The constructive nature of memory leads to a series of other errors that could also be tested in bees. For example, humans have been shown to misattribute the context in which particular information is acquired—so-called ‘source monitoring’ errors [61]. In this regard, recent work on source memory has shown that rats remember bound episodic memories—which include spatial information—for longer than spatial information on its own [8,9,62]. Future research assessing the different forgetting rates of multiple elements by bees may shed light on the binding of false integrated representations. Likewise, the conceptual similarity between two items (i.e. ‘relatedness’) has also been shown to lead to misremembering the presence of an item that was never experienced [63,64]. Memory interference—i.e. presentation of information that interferes with the retention of the to-be-remembered information [65,66]—is another phenomenon that could provide information on the reconstructive nature of memory. Thus, it would be useful to extend this line of research to these memory errors to further examine bees’ constructive memory. Given the present results, it would also be meaningful to examine bees’ abilities to remember spatio-temporal information [1]. This would allow us to establish whether bees’ memory resembles human memory in content.

To conclude, the results presented here show evidence of bees spontaneously making memory conjunction errors. The findings tantalizingly suggest the presence of constructive processes in bees’ memories. Memory error paradigms, like the one presented here, offer an interesting avenue of research to examine episodic memory from a novel approach since constructive processes can be used to combine and recombine elements of past events to imagine future ones. The comparative field of episodic memory is, therefore, ripe for being taken beyond our established paradigms and old debates, and into a more mature and constructive phase.

## Data Availability

Data are included in the electronic supplementary material [67].
